# Designing Personalization Cues for Museum Robots: Docent Observation and Controlled Studies

**DOI:** 10.3390/s25227095

**Published:** 2025-11-20

**Authors:** Heeyoon Yoon, Min-Gyu Kim, Sara Kim, Jin-Ho Suh

**Affiliations:** 1 Human-Robot Interaction Research Center, Korea Institute of Robotics and Technology Convergence, Pohang 37553, Republic of Korea; yuni@kiro.re.kr (H.Y.); mingyukim@kiro.re.kr (M.-G.K.); 2 Institute of Library, Information and Media Science, University of Tsukuba, Tsukuba 305-8550, Japan; kimsun@slis.tsukuba.ac.jp; 3 Major of Mechanical System Engineering, Pukyong National University, Busan 48513, Republic of Korea

**Keywords:** human–robot interaction, perceived personalization, knowledge alignment, visual recognition accuracy, preference inquiry, memory continuity, science museum

## Abstract

Social robots in public cultural venues, such as science museums, must engage diverse visitors through brief, one-off encounters where long-term user modeling is infeasible. This research examines immediately interpretable behavioral cues of a robot that can evoke a sense of personalization without storing or profiling individual users. First, a video-based observational study of expert and novice museum docents identified service strategies that enable socially adaptive communication. Building on these insights, three controlled laboratory studies investigated how specific cues from robots influence user perception. A video-based controlled study examined how recognition accuracy shapes users’ social impressions of the robot’s intelligence. Additional studies based on the Wizard-of-Oz (WoZ) method tested whether explanatory content aligned with participants’ background knowledge and whether explicit preference inquiry and memory-based continuity strengthened perceptions of personalization. Results showed that recognition accuracy improved social impressions, whereas knowledge alignment, explicit preference inquiry, and memory-based continuity cues increased perceived personalization. These findings demonstrate that micro-level personalization cues, interpretable within a short-term encounter, can support user-centered interaction design for social robots in public environments.

## 1. Introduction

Service robots are increasingly introduced into public environments such as museums, transportation hubs, and retail settings, where they must accommodate diverse users and enhance service satisfaction through both technical performance and well-designed human–robot interaction. In these contexts, interactions with robots are typically brief, unpredictable, and often non-repetitive, making personalization based on long-term user data difficult to achieve. Conventional approaches such as user modeling, adaptive learning, and preference histories are therefore limited in effectiveness when users remain anonymous and re-encounters are rare [1].

To address this challenge, robots should be designed to convey individualized responsiveness through immediately interpretable behavioral cues rather than data-intensive personalization. Context-sensitive actions, affective responsiveness, and well-timed interactional signals can function as key elements for delivering socially plausible, real-time personalization [2,3]. When carefully orchestrated, such cues can transform even momentary encounters into meaningful and memorable experiences [4]. These behavioral principles provide a foundation for designing social robots that appear adaptive and attentive without relying on stored personal information.

Building on this perspective, the present research focuses on four immediately interpretable cues that can be implemented within short, anonymous encounters: performance competence, adaptive explanations, explicit preference inquiry, and memory-based continuity. These four cues represent distinct dimensions of user-centered responsiveness—cognitive, informational, and relational. While each cue has been individually linked to personalization in data-driven contexts, its immediate effects on user perception have rarely been examined under short interactions. Moreover, few approaches have grounded such cue designs in the proven interactional expertise of human docents, who effectively tailor their communication to diverse visitors without long-term user modeling. Therefore, this study empirically examines how such immediately interpretable cues can foster perceived personalization—users’ recognition that the robot tailored its behavior to them—which is conceptually distinct from general positive impressions such as interest or comfort that may arise even without individualized adaptation.

To operationalize this framework, the research consisted of four complementary studies. Section 3.2 involved a comparative observation of experienced and novice science docents to identify communication patterns associated with service expertise. Drawing on these findings, Section 3.3 tested how a robot’s cognitive performance influenced users’ social impressions. Section 3.4 examined whether tailoring explanatory content to participants’ knowledge levels enhanced perceived personalization. Finally, Section 3.5 evaluated how explicit preference inquiry and continuity cues shaped users’ relational perceptions of the robot.

The remainder of this paper is structured as follows. Section 2 reviews related work on personalization in public service Human-Robot Interaction (HRI), contrasting data-driven methods with immediate, interpretable cues. Section 3 details the methods and hypotheses across four studies. Section 4 reports the results for each study, and Section 5 integrates these findings to derive design implications for scalable and privacy-aware personalization. Section 6 concludes with key contributions and directions for future research.

## 2. Related Works

### 2.1. Public Service HRI

Designing socially adaptive robots in public service domains often involves modeling human experts who successfully manage dynamic interpersonal contexts. Museum docents exemplify such expertise: they tailor explanations and conversational styles according to diverse visitor characteristics such as age, background knowledge, and communication preferences. Therefore, analyzing docent strategies offers practical insights for developing robotic docents.

Previous research in the HRI field has emphasized the importance of user-tailored adaptation for enhancing trust, likability, and sustained engagement. For example, it has been demonstrated that when a robot coordinates its dialogue in accordance with user expectations, both perceived trust and system acceptance increase significantly [5]. Similarly, the evidence suggests that robots adjusting response timing and utterances based on attentional cues contributed to higher levels of user interest, empathy, and overall satisfaction [6].

However, adaptive behaviors do not always guarantee positive outcomes. It has been reported that even when a robot switched its behavior or persona according to different users in a multi-user environment, there were no significant effects on interaction duration or user evaluations [7]. In some cases, such visible adaptations even disrupted the natural flow of interaction or undermined its coherence. Furthermore, it has been demonstrated that in high-stakes contexts such as asking participants to pick up a hot plate or offering food to those who were fasting, excessive display of social cues by the robot led to a decline in user trust [8]. Prior studies suggest that adaptive behavior tends to be interpreted more positively when users can infer a plausible intention or contextual relevance behind the change [9]. This indicates that personalization in real-world interactions should remain socially interpretable rather than opaque.

While adaptation is crucial for social robots, much of the existing personalization literature determines how to adapt by relying on stored user-specific information through long-term preference modeling, reinforcement learning based on repeated encounters, or affect-sensitive interaction histories [10,11,12]. These approaches inherently assume persistent user identification and extended engagement with identifiable individuals. However, museums often face challenges with personalization due to privacy protection requirements and practical scalability constraints, particularly limiting long-term, individualized data use [13].

Given these constraints, one promising approach is to use personalization cues that are immediately interpretable without accessing or storing personal data, enabling meaningful adaptation even in brief, anonymous encounters. Accordingly, this work investigates non-data-driven personalization cues that users can perceive and interpret within a short-term interaction. To ground these cues in established service practices, we begin by observing expert museum docent (Study 1) to extract adaptation strategies that are both feasible under privacy constraints and socially interpretable to visitors in public museum settings.

### 2.2. Personalization in HRI: Data vs. Immediate Cues

Visitor engagement in museum settings is closely linked to the richness of their cognitive experience. It is therefore strongly influenced by the extent to which explanatory content aligns with their prior knowledge [14]. Since one of the primary purposes of museum visits is learning, explanations must be both intellectually accessible and contextually appropriate [15]. Well-trained human docents can intuitively or conversationally assess each visitor’s level of understanding and naturally adjust the level and content of their explanations. Likewise, effective robotic docents should be able to provide meaningful and memorable experiences even in brief, one-off interactions [16].

When a robot’s explanations are sensitive to context and well attuned to visitors’ interests or focal attention, engagement is enhanced and perceptions of personalization are strengthened. Conversely, when explanations fail to align with visitors’ prior knowledge [17] or fail to respond to signals of disinterest [18], the experience is perceived as less personalized. Beyond cognitive alignment, however, effective personalization also depends on how accurately robots can identify and respond to visitors’ individual interests.

Docent robots support visitors by either directly asking about their interests or inferring them from observable characteristics. However, inaccurate inferences can lead to inappropriate recommendations, thereby undermining the robot’s effectiveness. To avoid such mismatches, it is important to explicitly verify visitors’ preferences in order to deliver personalized exhibition experiences effectively [19]. Prior research indicates that when systems infer user attributes, the level of user acceptance depends on the accuracy and sensitivity of the inferred data; when the inference is incorrect, agreement levels tend to decrease [20]. Therefore, even if a robot accurately identifies a visitor’s attributes, additional confirmation procedures are necessary to prevent mismatches and ensure that the interaction is perceived as genuinely personalized.

Within this context, the key factor that makes users experience interactions as personally meaningful lies in verification procedures that ensure alignment with their intentions. Previous studies have also reported that, in addition to input modalities, the design of confirmation procedures plays a critical role in shaping user preferences [19]. When robots provide confirmation processes in the form of explicit questions or selectable options, visitors are more likely to perceive that the content matches their intentions, thereby strengthening their preference for personalized services. In contrast, personalization strategies based solely on demographic assumptions risk producing misaligned recommendations, which can feel generic or even alienating in diverse public settings such as museums [21]. Strategies that present multiple alternatives and allow users to confirm their preferences have been shown to be effective [22]. Accordingly, implementing confirmation strategies in HRI not only reduces such risks but also fosters perceptions of the robot as responsive and cognitively attuned.

Although most interactions in exhibition environments are brief and one-off, visitors may, under certain conditions, encounter the same robot multiple times. For instance, a visitor may repeatedly meet the same robot while moving through different exhibition sections, participating in consecutive interactive programs, or resuming group interactions after short breaks. In these situations, personalization strongly depends on within-visit continuity of experience, with memory functions playing a central role. When a robot recalls a visitor’s previous behaviors, preferences, or conversation topics, it creates the impression that the interaction flows naturally rather than being fragmented [23]. Such continuity reinforces perceptions of social presence and personalization, making the visitor’s experience more coherent and user-centered. Conversely, when a robot fails to recall prior interactions, visitors may perceive each encounter as disconnected, thereby weakening the impression of personalized service [20].

However, personalization depends not only on whether a memory function is present but also on how it is expressed in interaction. Even minimal cues such as referring back to previously discussed exhibits, addressing the visitor by name, or acknowledging past choices can effectively manifest this continuity [13]. Empirical evidence further demonstrates that when robots explicitly recall and reference information from prior interactions, users interpret such personalized responses as indicators of higher social intelligence and adaptability [24].

Although knowledge alignment, preference verification, and memory continuity provide promising means of delivering personalization without long-term identification, little is known about the underlying cognitive mechanisms through which users interpret such cues as socially meaningful. Beyond these relational cues, performance-related behaviors may also serve as foundational signals that shape users’ expectations about a robot’s capacity for social attunement. This motivates a closer examination of how competence cues influence social perception.

Based on these considerations, this paper investigates how these three personalization strategies influence user perceptions through a series of user studies: Study 2 examines recognition competence as a basis for perceived personalization, Study 3 explores knowledge alignment through adaptive explanations, and Study 4 evaluates preference verification and memory continuity as mechanisms that construct personalized impressions in public robot interactions.

### 2.3. Social Cognitive Mechanisms Underlying Personalization

Perceived intelligence is a core dimension of HRI and plays a crucial role in shaping user engagement. It refers not merely to a robot’s ability to perform tasks effectively, but also to the social impression that leads users to interpret the robot as intentional and socially understanding. For example, it has been experimentally demonstrated that a robot’s functional performance is not limited to task success but is closely linked to users’ social evaluations such as trust, likability, and perceived human-likeness [25]. Extending this line of evidence, subsequent studies have shown that perceived intelligence and anthropomorphism jointly enhance trust and satisfaction [26]. Similarly, it has been shown that users’ perceptions of a robot’s competence significantly shape their preferences, exerting a more decisive influence than other factors [27]. Taken together, these findings suggest that users readily infer social attributes, such as likability and trust, based on cognitive evaluations.

Further evidence indicates that perceived performance during human–robot interaction plays a decisive role in users’ emotional experience [28]. Specifically, the higher the performance was perceived to be, the more users reported feeling comfortable, calm, and safe in the presence of the robot. Such emotional comfort contributes to a broader sense of psychological safety, suggesting that reliably executed tasks can provide reassurance beyond mere technical success.

Social cognition research further indicates that users interpret not only performance cues [29] but also knowledge alignment [30] and relational continuity [20] as indicators of shared understanding and relationship maintenance. In public environments such as science museums, where visitors typically encounter robots for the first time and engage in brief, spontaneous interactions, the importance of performance cues becomes even greater. In such contexts, robots have limited opportunities to adjust relationships through complex adaptive behaviors, as long-term personalization is difficult due to the lack of sufficient user data. Consequently, immediate but straightforward cognitive cues may serve as the primary factors shaping user impressions.

Together, these lines of work suggest that personalization in public-service HRI may emerge from multiple types of immediately interpretable social cues ranging from task competence to cognitive alignment and relational continuity. However, prior research has not systematically compared these cues within a unified framework nor validated their effects in scenarios modeled after museum interactions under anonymity and data constraints.

To bridge this gap, this work aims to determine how users cognitively interpret immediately available personalization cues in brief, public encounters with a museum robot. Specifically, we address the following research questions:

RQ1. Do competence cues enhance users’ social impressions of a museum robot?
RQ2. Does knowledge alignment increase perceived personalization in brief museum encounters?
RQ3. Do preference verification and memory cues strengthen relational personalization in repeated encounters?

## 3. Methods

Four studies were conducted to empirically examine the proposed framework across observational and experimental settings.

### 3.1. Hypotheses

Drawing from the literature reviewed in Section 1 and Section 2, we focused on immediately interpretable personalization cues and examined how each influences users’ social perceptions of a robot. Accordingly, the following hypotheses were proposed.

To begin, higher cognitive performance is expected to drive more favorable social attributions:

- **Hypothesis 1-1:** Users will attribute greater human-likeness to a robot with higher recognition accuracy.
- **Hypothesis 1-2:** Users will perceive a robot with higher recognition accuracy as more animated and lifelike.
- **Hypothesis 1-3:** Users will express higher affinity and emotional acceptance toward a robot with higher recognition accuracy.
- **Hypothesis 1-4:** Users will assess a robot with higher recognition accuracy as having higher cognitive competence.
- **Hypothesis 1-5:** Users will feel more secure and relaxed in the presence of a robot with higher recognition accuracy.

In addition, personalization is expected to diminish when a robot’s explanations fail to reflect the user’s background:

- **Hypothesis 2:** When a robot’s explanation is misaligned with a visitor’s background knowledge, the perceived personalization of the service will be significantly reduced.

Furthermore, failing to elicit or maintain personalized information accurately is expected to undermine personalization perceptions:

- **Hypothesis 3:** When a robot delivers personalized services based solely on inferred user preferences without explicit inquiry, the perceived personalization of the service will be significantly reduced.
- **Hypothesis 4:** When a robot fails to demonstrate memory of a user’s prior interaction (e.g., name, past choices, or preferences), the perceived personalization of the service will be significantly reduced.

### 3.2. Study 1: Observation of Human Service Behavior as Design Basis

#### 3.2.1. Participants

Study 1 involved two museum docents, one novice with less than 1 year of experience, and one experienced with more than 4 years of experience, for video-based observation. In addition, a follow-up interview was conducted with seven more docents who had participated in the behavioral observations.

#### 3.2.2. Measures

We developed a structured taxonomy of docents’ behaviors comprising two primary categories for behavior coding, service-level behavioral units and basic action units. The service-level behavioral units captured observable patterns of interaction within distinct service episodes and were grouped into three dimensions: flexibility, proactivity, and interpersonal skills. Each definitions for these service behavior categories are provided in Table 1.

The basic action units were organized into four categories: gaze, move, point, and talk, with definitions as presented in Table 2.

Through the behavioral observations, overt actions such as gaze, pointing, movement, and speech were identified. These offered limited insight into the cognitive processes and situational judgments underlying such behaviors. Therefore, to gain a deeper understanding of docent behavior, the follow-up interviews were designed to explore how docents perceive visitor characteristics and adjust their communicative strategies accordingly. In particular, the questions focused on how and why docents estimate visitors’ knowledge levels, modify their explanatory styles, and elicit personalized interactions. The questions used in the follow-up interview are presented in Table 3.

#### 3.2.3. Data Collection Procedure

Data collection for video observations was conducted at the RoboLife Museum located in Pohang, South Korea. Interaction scenes between visitors and the docents were collected as video data. Upon entering the exhibition hall, visitors were informed of the purpose of data collection, its intended use, and the security measures in place, after which written consent was obtained. For child participants, written consent was obtained from their legal guardians. The videos were recorded as the docent followed the tour path to capture both the docent’s explanations and visitors’ behavior throughout the exhibition space. A follow-up interview was then conducted to explore further the reasoning and communication strategies underlying their externally observable behaviors. The interviews were conducted in a semi-structured format, with the interpreters answering questions posed by the interviewer as presented in Table 3. The detailed observation and interview results are described in Section 4.1.

### 3.3. Study 2: Cognitive Performance Cues and Social Perception

#### 3.3.1. Participants

Study 2 recruited 31 adults (*M* = 33.42 years, *SD* = 7.49), consisting of 10 males and 21 females.

#### 3.3.2. Measures

The participants’ subjective evaluations of the robot were collected using validated self-report questionnaires on a 5-point Likert scale (1 = Strongly Disagree, 5 = Strongly Agree), completed immediately after the interaction. Study 2 employed the Godspeed scale to assess five core dimensions of human-robot interaction [31].

#### 3.3.3. Stimuli

Two video stimuli were used in which the robot’s visual recognition accuracy was manipulated as the experimental condition. In each video, a human partner sequentially presented ten picture cards to the robot, and the robot verbally responded to each card based on its recognition output. In the first video, the robot’s recognition accuracy was set to approximately 50%, whereas in the second video, the accuracy was increased to 80% while maintaining the same interaction structure and scripted content. All aspects of the interaction were held constant across the two videos, with only the accuracy level being manipulated between conditions.

The robot was used with Pibo (Circulus Co., Ltd., Seoul, Republic of Korea). It is a companion-type social robot equipped with 10 degrees of freedom (two motors each for the head, arms, hands, legs, and feet), enabling basic gestural expression. The robot features a 128 × 64 OLED display, a 5-MP CMOS camera, dual 5Wspeakers, a MEMS microphone, and touch and human-detection sensors, as well as RGB LEDs in the eyes to support multimodal interaction. Its physical specifications are 250 mm × 395 mm × 125 mm and 2.2 kg. The robot’s speech was produced using Google Text-to-Speech (TTS), and its gestures were executed through pre-defined motion scripts synchronized with each utterance. The robot’s expressions were remotely controlled via a PC interface over Wi-Fi, triggering preset speech–gesture pairs to ensure consistent behavior across participants.

#### 3.3.4. Procedure

The video-based methodology was adopted based on prior evidence demonstrating its high ecological validity and strong correspondence with live interaction outcomes [32]. Using a within-subjects design, each participant viewed two prerecorded video clips in which Pibo interacted with a human partner who sequentially presented ten picture cards. Pibo verbally responded to each card based on its recognition results. The only manipulated factor was visual recognition accuracy (50% vs. 80%), while the script, timing, and interaction sequence remained identical. After viewing each clip, participants completed a questionnaire assessing their impressions of the robot, and the order of the two accuracy conditions was counterbalanced. The results about the effect of cognitive performance on social impression are elaborated in detail in Section 4.2.

### 3.4. Study 3: Knowledge Alignment and Perceived Personalization

#### 3.4.1. Participants

30 university students (*M* = 23.20 years, *SD* = 1.97) were included, comprising 7 males and 8 females from engineering majors and 4 males and 11 females from non-engineering majors. The participants were recruited from the undergraduate population at Handong Global University, South Korea, and they indicated whether they had an engineering or non-engineering background on the questionnaire.

#### 3.4.2. Measures

Study 3 evaluated perceptions of personalization using measures of perceived personalization [33], perceived care [34], and user satisfaction [35], along with the Godspeed questionnaire subscales [31]. Responses were collected using a 5-point Likert scale (1 = Strongly Disagree, 5 = Strongly Agree).

After the interaction, the participants were asked to write a brief summary of the robot’s explanation, which served as a measure of how well they understood the content. A content coverage score was calculated based on the presence of four pre-defined technical terms embedded in the explanation. Although this measure primarily captured the participants’ comprehension, it also functioned as an ex-post manipulation check by confirming that the engineering and non-engineering groups differed in their understanding as intended.

#### 3.4.3. Stimuli

The participants interacted with Pibo, a companion-type social robot, while knowledge alignment was manipulated as the experimental condition. To operationalize this manipulation, Pibo provided explanations that deliberately incorporated technical robotics terminology. The explanatory content was designed for an upper-level undergraduate background in engineering disciplines, and the script was developed by junior- and senior-level robotics majors from Handong Global University.

The explanations delivered by Pibo included terms such as SSH (Secure Shell), bounding box, PD (Proportional-Derivative) control, and STT (Speech-to-Text), reflecting the knowledge level typically expected of undergraduate engineering students. For example, SSH was introduced as: “I operate through programming via remote access using SSH and exchange information between a server and a local system through socket communication.” Bounding box was presented in the context of visual tracking: “I can establish eye contact with individuals by calculating the x and y coordinates of the center of the bounding box for the target person in the image and adjusting my neck joint motors accordingly.” PD control was described as follows: “To achieve smooth and natural neck movements, PD control is applied. By adjusting the P and D values, I can regulate motor speed for fluid motion.” Lastly, STT was explained as: “I can engage in conversation with users by using the Google Cloud Platform’s STT-API to recognize spoken words.”

#### 3.4.4. Procedure

A between-subjects design was applied using a WoZ method, where Pibo’s utterances were remotely controlled to simulate fully autonomous execution. Pibo initiated a short greeting and then delivered a scripted explanation describing its internal mechanisms while incorporating domain-specific technical terminology. The participants listened attentively to the full explanation, summarized the content immediately afterward in writing, and then completed the questionnaire evaluating their perceptions of the interaction. The detailed results about the effect of knowledge alignment on perceived personalization and social impression are presented in Section 4.3.

### 3.5. Study 4: Preference Inquiry and Memory Continuity

#### 3.5.1. Participants

42 adults (*M* = 23.14 years, *SD* = 1.72) were involved, including 20 males and 22 females.

#### 3.5.2. Measures

Study 4 focused on constructs related to personalized service perception, using perceived personalization [33], perceived care [34], and user satisfaction [35]. Responses were collected using a 5-point Likert scale (1 = Strongly Disagree, 5 = Strongly Agree).

#### 3.5.3. Stimuli

In Study 4, the participants interacted with Pibo, while two personalization strategies were manipulated: explicit preference inquiry (asking vs. inferring) and memory continuity (remembering vs. forgetting). As part of the preference manipulation, four sticker designs were created as personalized reward stimuli and grouped into two stylistic categories. Two stickers represented a cute aesthetic, characterized by rounded shapes and warm, childlike features (e.g., a small mouse in a dress and a cat with a bow), while the other two displayed a stylish aesthetic with bold, angular designs and action-oriented themes (e.g., a heroic emblem and an adventurous mascot). These stylistic contrasts were selected because such visual cues are commonly associated with gender-based preference assumptions, allowing comparison between preference inference and explicit inquiry conditions. All stickers were matched in size (approximately 10 cm × 10 cm), material, and print quality to ensure that only stylistic attributes varied. Additionally, memory-based personalization was tested by having Pibo either recall or fail to recall each participant’s name and previously selected sticker during a subsequent session.

#### 3.5.4. Procedure

A between-subjects design was implemented across two sessions, also using a WoZ method to allow the robot to appear fully autonomous during dialogue and reward presentation. The experiments took place over four weeks, randomly assigned to two conditions in the first session (asking preference):

- Preference-asking group (*n* = 22): The robot explicitly asked for participants’ sticker preferences prior to service delivery.
- Preference-inferring group (*n* = 22): The robot inferred preferences based on gender-based preference assumptions.

Each participant engaged in a structured interaction consisting of three phases:

- Introduction Phase: The robot greeted the participant, asked for their name, and (in Preference-asking group only) asked which type of sticker they preferred cute or stylish.
- Task Phase: The robot introduced itself, explained its capabilities (e.g., visual tracking, STT, system architecture), and maintained rapport through gaze and responsive dialogue.
- Reward Phase: In Preference-asking group, the robot delivered the participant’s chosen sticker. In Preference-inferring group, the robot delivered a sticker based on internal logic without asking for preferences.

In the second session (remembering users), only participants from Preference-asking group were re-invited. After excluding two participants who withdrew, a total of 20 participants took part in the second session. These participants were then divided into the following subgroups:

- Remember-user group (*n* = 10): The robot remembered the participant’s name and previous preference, greeting them accordingly and using this information during the reward phase.
- Forget-user group (*n* = 10): The robot failed to recall the prior interaction, asked for the name again, and re-queried the sticker preference.

This second session involved a creative task in which participants were asked to draw and describe a memorable experience. The robot responded empathetically to participants’ explanations and, during the reward phase, either recalled the previously selected sticker (Remember-user group) or re-queried it (Forget-user group), delivering the reward accordingly. After each session, the participants completed the same survey measuring their perceptions of personalization, care, and usefulness. The results about the effects of explicit preference inquiry and memory-based continuity on perceived personalization are reported in Section 4.4.

## 4. Results by Individual Studies

### 4.1. Study 1: Observation of Human Service Behavior as Design Basis

The analysis was manually performed using ELAN [36] software 6.2 based on the behavior coding schemes shown in Table 1 and Table 2. ELAN was selected for its ability to support fine-grained, frame-by-frame annotation of multimodal behaviors, which was essential for capturing the nuanced gaze, gestures, and verbal-nonverbal coordination observed in docent–visitor interactions. This manual method was preferred over automated video analysis tools, which may lack the sensitivity to detect these subtle but socially significant behaviors. The results revealed notable differences between expert and novice groups in both the count (times) and duration (ms) of behaviors.

#### 4.1.1. Differences in Service-Level Behaviors Between Novice and Expert

**Figure 1:**
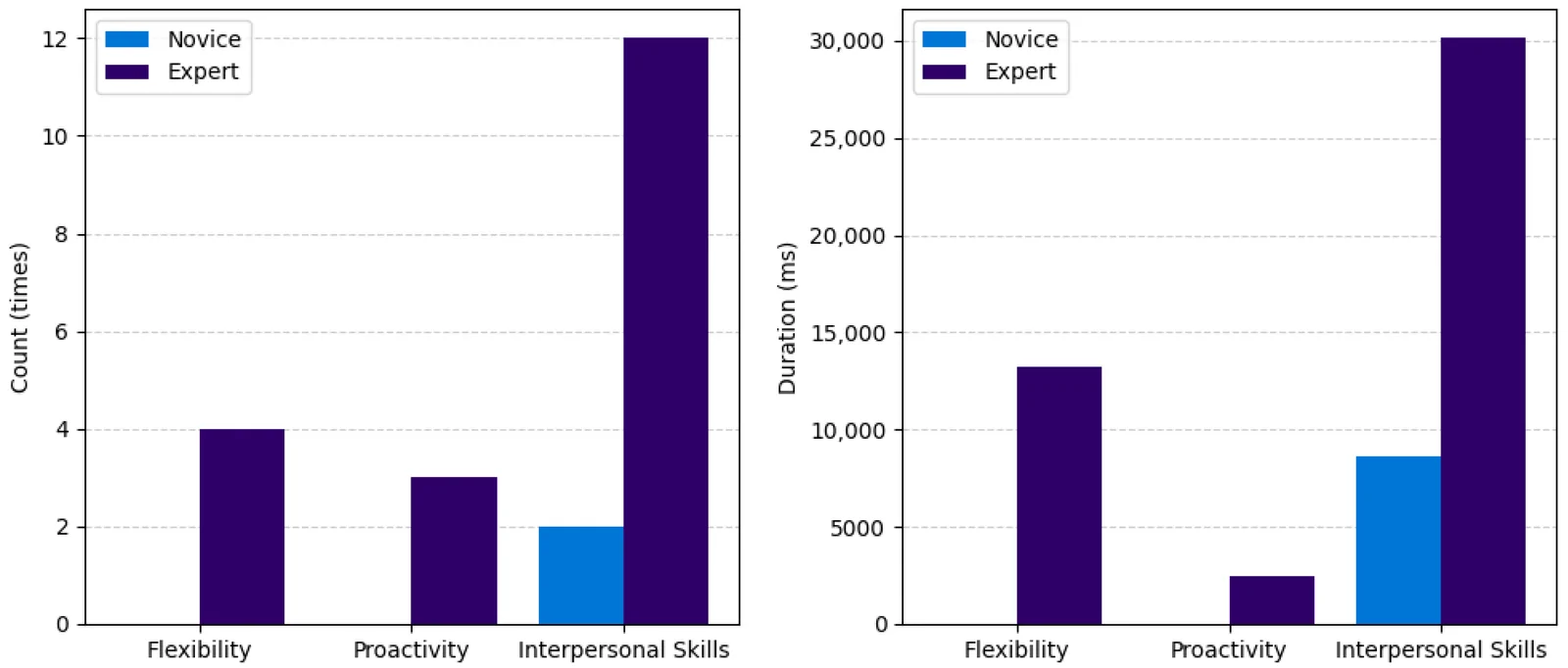
Differences in service-level behaviors between the novice and the expert docents.

A comparison of service-level behaviors between expert and novice docents revealed differences even in higher-level interaction strategies. The results are illustrated in Figure 1. In terms of flexibility, the expert exhibited this behavior four times, with a total duration of 13,261 ms, whereas this was not observed in the novice. The expert’s flexibility was evident in situations where the complexity or delivery style of explanations was immediately adjusted, based on cues such as the visitor’s age, the predicted level of understanding, or the visitor’s attentiveness. On the other hand, the novice tended to maintain a uniform delivery of explanations and showed little effort to adapt to visitors’ changing responses.

Proactivity, defined as anticipatory behavior that addresses visitor needs before they are explicitly expressed, was also observed only in the expert, occurring three times with a total duration of 2464 ms. An illustrative case was when the expert noticed children struggling to see the exhibits because adults blocked them and proactively guided them to move to the front.

The most significant difference was found in interpersonal skills, specifically in communicative behaviors such as speech and gestures directed toward visitors. The expert demonstrated such behaviors 12 times, totaling 30,117 ms, whereas the novice displayed them only twice, with a total duration of 8663 ms. This contrast suggests that the expert employed a broader range of interaction strategies, such as attending to subtle visitor reactions and actively directing attention to the exhibits, although the novice used these skills only occasionally.

#### 4.1.2. Differences in Basic Actions Between Novice and Expert

**Figure 2:**
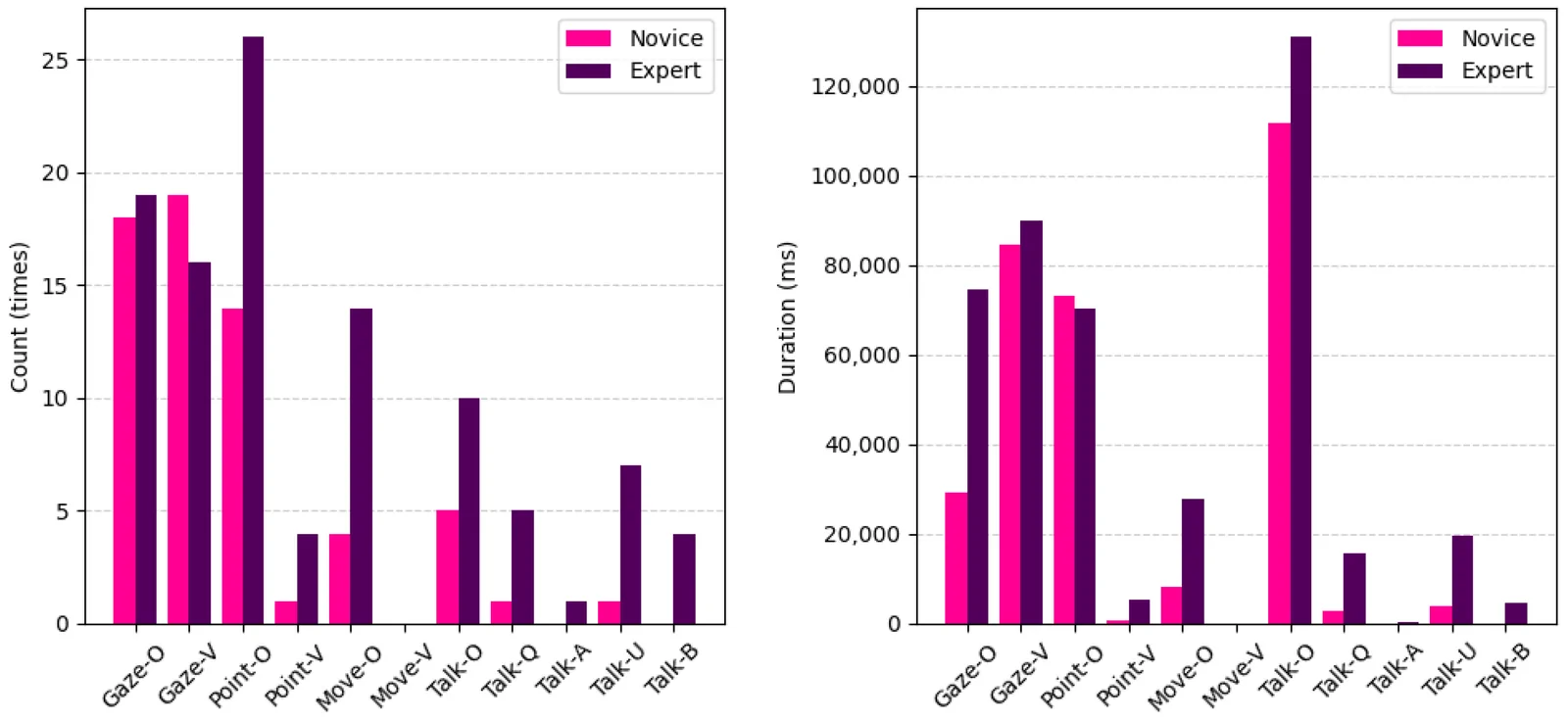
Difference in basic actions between the novice and the expert docents.

As depicted in Figure 2, in terms of visual attention, object-gazing (Gaze-O) occurred 19 times for the expert and 18 times for the novice, showing similar frequencies. However, the cumulative duration for the expert (74,619 ms) was more than twice that of the novice (29,343 ms), suggesting that the expert maintained more deliberate and sustained attention to the exhibits. Visitor-gazing (Gaze-V) occurred 16 times for the expert and 19 times for the novice, with total durations also comparable (89,833 ms vs. 84,665 ms). Overall, the expert showed similarly long durations for both object-gazing and visitor-gazing. In contrast, the novice devoted more than twice as much time to visitor-gazing compared to object-gazing. It indicates that the expert regarded both object and visitor attention as essential components of interaction, whereas the novice primarily maintained visual engagement with visitors and briefly shifted her gaze to exhibits when explaining them.

Regarding gestural behaviors, object-pointing (Point-O) occurred 26 times for the expert (70,401 ms) and 14 times for the novice (73,261 ms). The expert initiated pointing gestures more frequently, while the novice tended to sustain them slightly longer, presumably to secure visitor comprehension. Visitor-pointing (Point-V) showed a more apparent distinction: the expert performed this gesture four times (5495 ms), whereas the novice did so only once (630 ms). It suggests that the expert used visitor-pointing strategically to engage visitors directly during explanations.

Locomotive behaviors also differed between the expert and the novice. Moving toward objects (Move-O) was observed 14 times for the expert (27,687 ms) and 4 times for the novice (8063 ms), indicating that the expert more frequently repositioned themselves to facilitate object-centered explanations or demonstrations. Moving toward visitors (Move-V) was not observed in either docent, suggesting that directly approaching visitors was not employed as a strategy in this context.

Verbal behaviors further highlighted the differences. Utterances about objects (Talk-O) were observed 10 times for the expert (130,925 ms) and 5 times for the novice (111,878 ms), confirming that object-related explanations were central for both. However, questions (Talk-Q) were produced more actively by the expert (5 times, 15,738 ms) compared to the novice (1 time, 2906 ms), indicating that the expert more frequently used questions to encourage visitor participation. Answers (Talk-A) occurred only once in the expert (420 ms), reflecting the docent’s response to a visitor’s question during explanation. Given the low frequency and short duration of both questions and answers, verbal exchanges between docents and visitors were relatively limited. Utterances related to unexpected situations (Talk-U) were more frequent in the expert (7 times, 19,565 ms) than in the novice (1 time, 3797 ms). For example, the expert explained the principle behind how a robot could stand up again after falling during a demonstration, or clarified that an insufficient power supply caused a robot to stop functioning and necessitated replacement. These instances demonstrate that the expert continued her explanations flexibly, even in unanticipated circumstances. Finally, backchannel cues (Talk-B) were observed only in the expert (4 times, 4730 ms), indicating that the visitors actively attempted to respond to the expert docent during interactions.

Taken together, the expert exhibited a broader behavioral repertoire and engaged more actively in sustaining explanations and initiating interaction with the visitors. In particular, behaviors such as visitor-pointing and questioning were coupled with visitors’ active responses, including backchanneling, demonstrating strategic interaction patterns. These behaviors were characterized by both higher frequency and longer duration, showing that the expert employed more effective strategies than the novice in eliciting and maintaining visitor engagement.

#### 4.1.3. Interview-Based Analysis

The docents who participated in the follow-up interviews reported frequently adjusting the complexity and tone of their explanations based on the perceived age and knowledge level of the audience. One docent noted, “When children come, I try not to use technical words. I sometimes ask their age directly and then adjust my explanation accordingly.” This strategy was especially important given the high frequency of child visitors, who were described as inquisitive, lacking prior domain knowledge: “Children ask a lot of questions, but they’re usually not based on what they’ve already learned.”

In terms of communication style, docents adapted both verbal and non-verbal behavior to suit younger audiences. One stated, “I use simpler vocabulary and more gestures when talking to kids.” Conversely, they occasionally encountered adults who asked more technical questions, prompting a shift in their delivery: “When someone asks something specific, like a working mom or a man who seems to know the topic, I switch to a more technical explanation.” These observations align with behavioral data indicating that experienced docents demonstrated greater verbal variety and adaptive gaze and gesture control, suggesting that personalized communication may be a hallmark of skilled service delivery.

In the lobby area, personalized service opportunities were more limited but still present. A docent described one such case: “If someone is carrying a baby, I sometimes tell them where the diaper-changing station is without them asking.” While this behavior was not universally observed, such proactive adjustments reflect a sensitivity to contextual cues, which may be considered the initial layer of personalization. Other tasks, such as guiding visitors to lecture rooms or restrooms, were more procedural. However, some docents attempted to add a personal touch through differentiated greetings: “I say ‘Hello!’ cheerfully to kids, but I use ‘Welcome’ or ‘Good day’ for adults.”

Although these practices may seem minor, they demonstrate the docents’ awareness of the social dynamics within the space and their attempts to align behavior with user needs subtly. This finding complements the service-level behavioral results, where experienced docents showed higher levels of proactivity and interpersonal skills, both of which are foundational to building user-centered, interactive service robots.

### 4.2. Study 2: Cognitive Performance Cues and Social Perception

**Figure 3:**
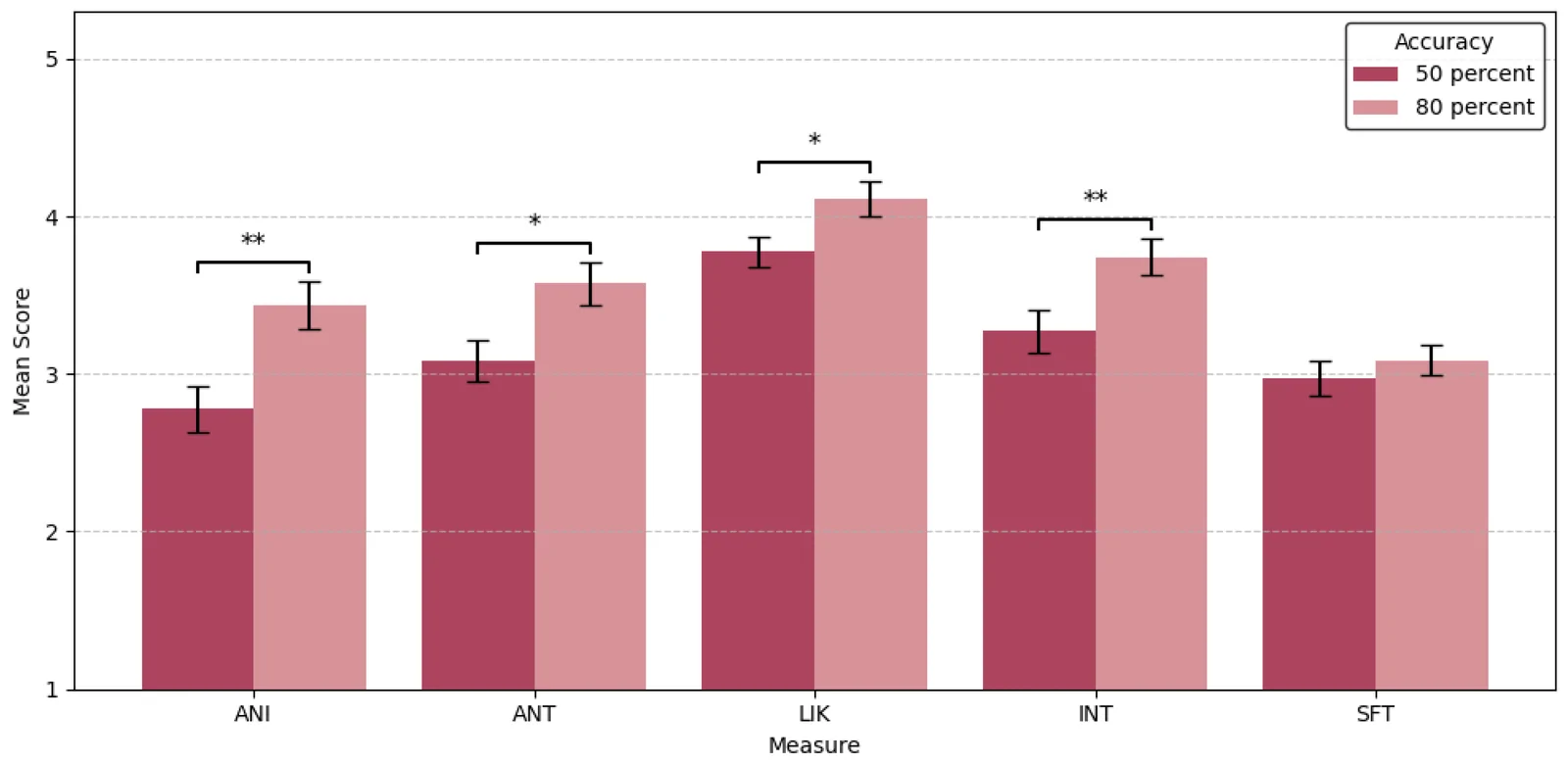
Wilcoxon signed-rank test results according to social impression for robot performance (ANI: Animacy, ANT: Anthropomorphism, LIK: Likeability, INT: Perceived Intelligence, and SFT: Perceived Safety). * *p* < 0.05, ** *p* < 0.01.

A Wilcoxon signed-rank test was conducted to examine whether recognition accuracy (80% vs. 50%) produced significant differences in anthropomorphism, animacy, likability, perceived intelligence, and perceived safety. This non-parametric test was selected due to the within-subjects design and the results of normality assessments. Specifically, Shapiro–Wilk tests revealed that several variables exhibited non-normal or borderline distributions, with p-values ranging from 0.060 to 0.097 in key measures such as anthropomorphism (80%) and perceived safety (80%). Given the modest sample size (*n* = 31) and sensitivity of the analysis to these distributional assumptions, the Wilcoxon test provided a robust and appropriate method for detecting within-subject differences without relying on parametric assumptions. The results are shown in Figure 3.

Consistent with the notion that minimal cognitive cues can evoke broader social attributions, the participants rated the robot as significantly more anthropomorphic when its recognition accuracy was 80% (*M* = 3.41, *SD* = 0.85) than when it was 50% (*M* = 2.69, *SD* = 0.82), *Z* = −3.035, *p* = 0.002. Despite the limited social behaviors or expressions, the participants inferred a higher degree of human-likeness purely from high task performance. This supports the idea that basic functional cues, such as visual recognition accuracy, can trigger spontaneous attribution of relational capacities. The result supports Hypothesis 1-1.

Similarly, the perceived animacy of the robot, defined as the extent to which it was seen as lifelike and autonomous, was significantly higher in the 80% condition (*M* = 3.57, *SD* = 0.75) compared to the 50% condition (*M* = 3.11, *SD* = 0.75) (*Z* = −2.438, *p* = 0.015). This indicates that higher task performance can alter users’ perception of the robot as a more self-directed and animated agent, reinforcing the role of intelligence cues in driving social cognition. The result supports Hypothesis 1-2.

The participants also reported significantly higher levels of likability toward the robot in the high-accuracy condition (*M* = 4.11, *SD* = 0.63) than in the low-accuracy condition (*M* = 3.77, *SD* = 0.53) (*Z* = −2.810, *p* = 0.005). This suggests that the participants associate higher task reliability with greater emotional affinity. The result supports Hypothesis 1-3.

As expected, perceived intelligence, defined as the robot’s cognitive capability as inferred by users, was significantly higher in the 80% accuracy condition (*M* = 3.74, *SD* = 0.65) than in the 50% condition (*M* = 3.27, *SD* = 0.77) (*Z* = −2.628, *p* = 0.009). This validates the effectiveness of recognition accuracy as a salient indicator of perceived intelligence, confirming that the participants readily interpret technical success as indicative of greater cognitive capability. The result supports Hypothesis 1-4.

Contrary to expectations, perceived safety did not significantly differ between the high-accuracy (*M* = 3.09, *SD* = 0.54) and low-accuracy (*M* = 2.97, *SD* = 0.62) conditions, *Z* = −1.042, *p* = 0.297. The result does not support Hypothesis 1-5. Given that the task involved a brief, low-risk, video-based observation, recognition accuracy alone may not have provided sufficient contextual cues to affect perceived safety.

### 4.3. Study 3: Knowledge Alignment and Perceived Personalization

#### 4.3.1. Group Difference in Perceived Personalization

**Figure 4:**
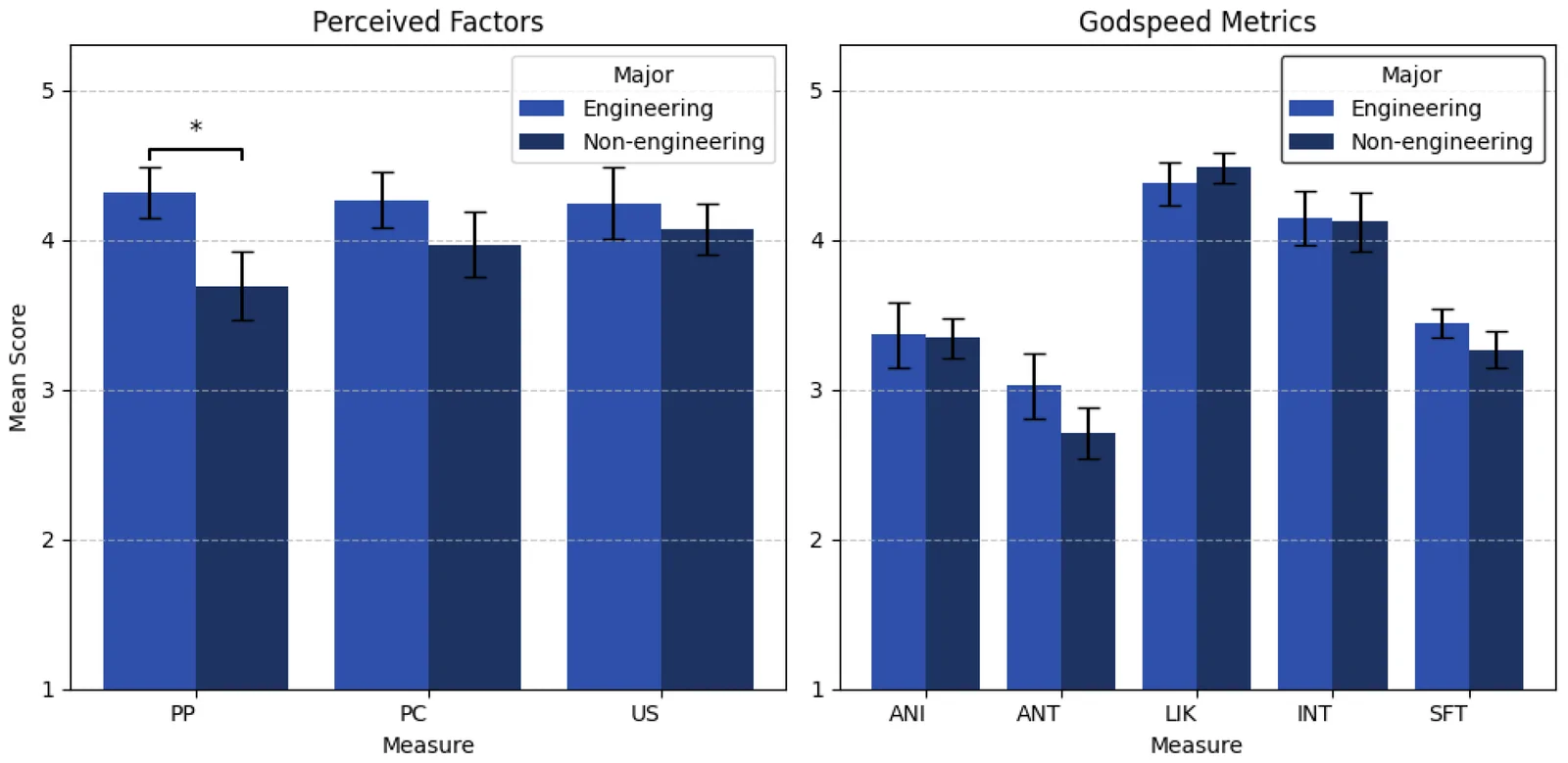
Mann-Whitney test results according to perceived personalization and social impression (PP: Perceived Personalization, PC: Perceived Care, US: User Satisfaction; ANI: Animacy, ANT: Anthropomorphism, LIK: Likeability, INT: Perceived Intelligence, and SFT: Perceived Safety). * *p* < 0.05.

A Mann–Whitney U test was conducted to examine whether differences in the participants’ background knowledge (Engineering vs. Non-Engineering) led to significant differences in perceived personalization, perceived care, and social impressions when interacting with the robot. This non-parametric test was selected due to the between-group comparison design and the outcome of normality tests, which indicated non-normal or borderline distributions in several variables. Notably, Shapiro–Wilk *p*-values fell below or near the 0.05 threshold for measures such as perceived care (Engineering: *p* = 0.023), user satisfaction (Engineering: *p* = 0.005), and likeability in Godspeed (Non-Engineering: *p* = 0.011). Given the small group sizes (*n* = 15 per group) and sensitivity of parametric tests to assumption violations in such contexts, the Mann–Whitney test was deemed a statistically robust and appropriate method for identifying group differences. The results are shown in Figure 4 (left).

A statistically significant difference in perceived personalization was found between the participants from engineering (*M* = 4.31, *SD* = 0.66) and non-engineering backgrounds (*M* = 3.69, *SD* = 0.89), *U* = 63.00, *Z* = −2.077, *p* = 0.038. The rank-biserial correlation *R* was −0.379, indicating a medium effect size. These results support the hypothesis that when the robot’s explanation incorporates domain-specific language aligned with the participant’s background knowledge, perceived personalization increases. Conversely, for the participants lacking such background knowledge, the robot’s explanation appears less tailored, resulting in reduced personalization. Thus, Hypothesis 2 was supported for perceived personalization, but not for other dependent variables.

No statistically significant difference was found in perceived care between the two groups (Engineering: *M* = 4.27, *SD* = 0.73; Non-engineering: *M* = 3.97, *SD* = 0.85) (*U* = 91.00, *Z* = −0.915, *p* = 0.360), with a small effect size (*R* = −0.167). This suggests that alignment with background knowledge had minimal impact on perceived emotional warmth or caring.

No significant group differences were found in user satisfaction (Engineering: *M* = 4.24, *SD* = 0.93; Non-engineering: *M* = 4.07, *SD* = 0.66) (*U* = 87.50, *Z* = −1.064, *p* = 0.287), with a small effect size (*R* = −0.194), indicating a weak association between knowledge alignment and overall satisfaction.

Mann-Whitney test results for the subscales of the Godspeed questionnaire revealed no statistically significant differences between groups, as shown in Figure 4 (right). For Animacy (Engineering: *M* = 3.37, *SD* = 0.84; Non-engineering: *M* = 3.34, *SD* = 0.52), the test yielded *U* = 95.00, *Z* = −0.729, and *p* = 0.466, with a small effect size (*R* = −0.133). Anthropomorphism (Engineering: *M* = 3.03, *SD* = 0.84; Non-engineering: *M* = 2.71, *SD* = 0.83) showed *U* = 84.50, *Z* = −1.166, and *p* = 0.243, also with a small effect (*R* = −0.213). Likeability (Engineering: *M* = 4.37, *SD* = 0.56; Non-engineering: *M* = 4.48, *SD* = 0.41) was not significantly different (*U* = 103.50, *Z* = −0.380, *p* = 0.704), with a very small effect size (*R* = −0.069). Perceived Intelligence (Engineering: *M* = 4.15, *SD* = 0.71; Non-engineering: *M* = 4.12, *SD* = 0.77) likewise showed no difference (*U* = 109.50, *Z* = −0.125, *p* = 0.900), with a negligible effect (*R* = −0.023). Finally, Perceived Safety (Engineering: *M* = 3.44, *SD* = 0.37; Non-engineering: *M* = 3.27, *SD* = 0.47) was also non-significant (*U* = 86.50, *Z* = −1.108, *p* = 0.268), with a small effect (*R* = −0.202). Overall, social impressions of the robot did not vary meaningfully based on background knowledge.

These findings show that while general impressions of the robot’s social attributes remained stable across groups, the alignment of explanation content with the participants’ prior knowledge influenced perceived personalization, supporting the hypothesis that knowledge mismatch can diminish the participant’s sense of being personally addressed. Therefore, Hypothesis 2 was supported only for perceived personalization, while other outcome measures did not show significant differences.

#### 4.3.2. Group Difference in Understanding

In addition to the survey results, the participants’ written responses were analyzed to evaluate their comprehension of the robot’s explanation. Engineering majors frequently incorporated discipline-specific terminology in their responses, demonstrating a high level of alignment with the robot’s explanation. In contrast, the non-engineering participants primarily recalled the content using their own linguistic frameworks, often omitting or paraphrasing the technical elements presented in the original explanation.

To quantitatively assess the participants’ level of understanding, a content coverage analysis was conducted. The analysis focused on identifying the presence of pre-defined technical terms in the participants’ responses and comparing the distribution of term coverage across the two groups. Four key technical terms were selected as analysis targets: SSH, bounding box, PD control, and STT. These were chosen based on their functional relevance to the robot’s system architecture, perceptual mechanisms, control theory, and interaction technologies.

For each participant, an unweighted content coverage score was calculated, representing the number of distinct terms accurately mentioned in their response. Each term was counted only once, regardless of repetition, in order to assess not how many times a specific term was written but rather how many different technical terms the participant actually knew and was able to use appropriately. The content coverage score was computed according to the following formula.

(1) $${\mathrm{Coverage}}_{\mathrm{unweighted}}\left(r\right)=\sum_{i=1}^{N}I\left(c_{i}\in r\right)$$

In Equation (1), ${\mathrm{Coverage}}_{\mathrm{unweighted}}\left(r\right)$ denotes the total number of key concepts identified in a given response *r*. The summation iterates over a pre-defined set of *N* key concepts, which in this study included: SSH, boundary box, PD control, and STT. $I(c_{i}\in r)$ is an indicator function that returns 1 if the ith concept $c_{i}$ is present in the response *r*, and 0 otherwise.

Descriptive analysis of the content coverage scores revealed clear differences between the two groups. The average unweighted coverage score for the engineering participants was 0.39, with 27.8% (10 out of 36) including at least one of the pre-defined technical terms in their written responses. In contrast, the average score for the non-engineering participants was markedly lower at 0.09, with only 8.7% (2 out of 23) referencing any technical term. The maximum score observed in the engineering group was 3, whereas no participant in the non-engineering group mentioned more than one term. These findings suggest that the engineering participants recalled a broader range of terms and understood the robot’s explanation in a manner more consistent with their domain-specific background knowledge.

**Figure 5:**
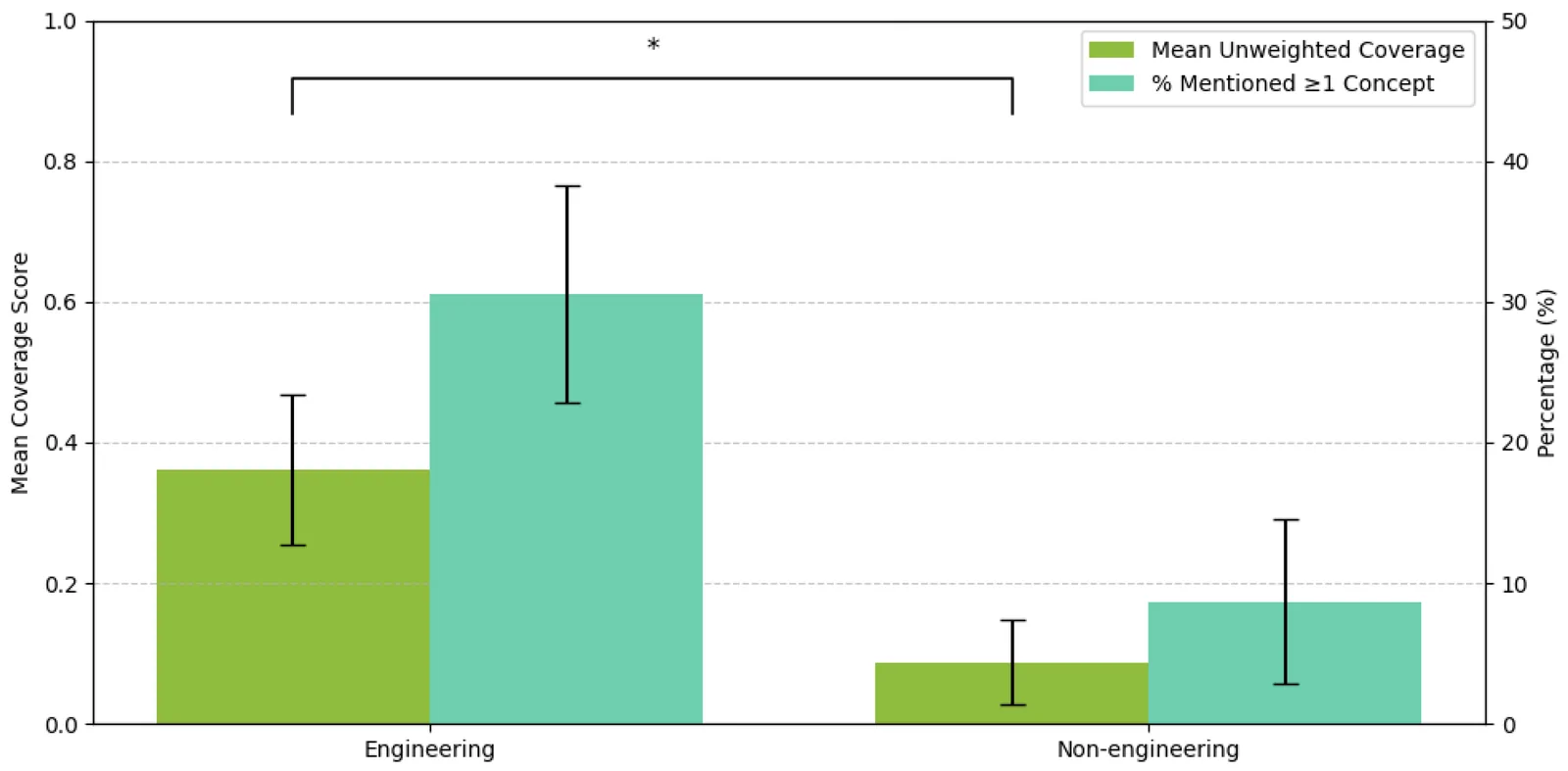
Content coverage score by group. * *p* < 0.05.

To examine the statistical significance of the group difference, a Mann–Whitney U test was conducted. The result indicated a statistically significant difference in content coverage scores between the two groups (*U* = 505.5, *p* = 0.0494). The results of the content coverage analysis are shown in Figure 5.

### 4.4. Study 4: Preference Inquiry and Memory Continuity

To examine the effects of the robot’s service delivery style—specifically whether it actively asked for user preferences (Preference-asking) and whether it remembered prior user information (Remember-user)—Mann–Whitney U tests were conducted on perceived personalization, perceived care, and user satisfaction. This non-parametric approach was selected due to the between-group design and evidence of non-normality in multiple key measures based on Shapiro–Wilk tests. In the preference-asking comparison, Shapiro–Wilk *p*-values were below 0.05 for most variables, including perceived personalization (*p* = 0.003), perceived care (*p* = 0.008), and user satisfaction (*p* = 0.004). Similarly, in the remember-user comparison, significant deviations from normality were observed for perceived personalization (*p* = 0.003) and perceived care (*p* = 0.001) in the remember-user group. These results justified the use of Mann–Whitney tests, which do not assume normal distributions and are robust for small sample sizes (*n* = 10–21 per group).

#### 4.4.1. Personalization Through Explicit Preference Inquiry

**Figure 6:**
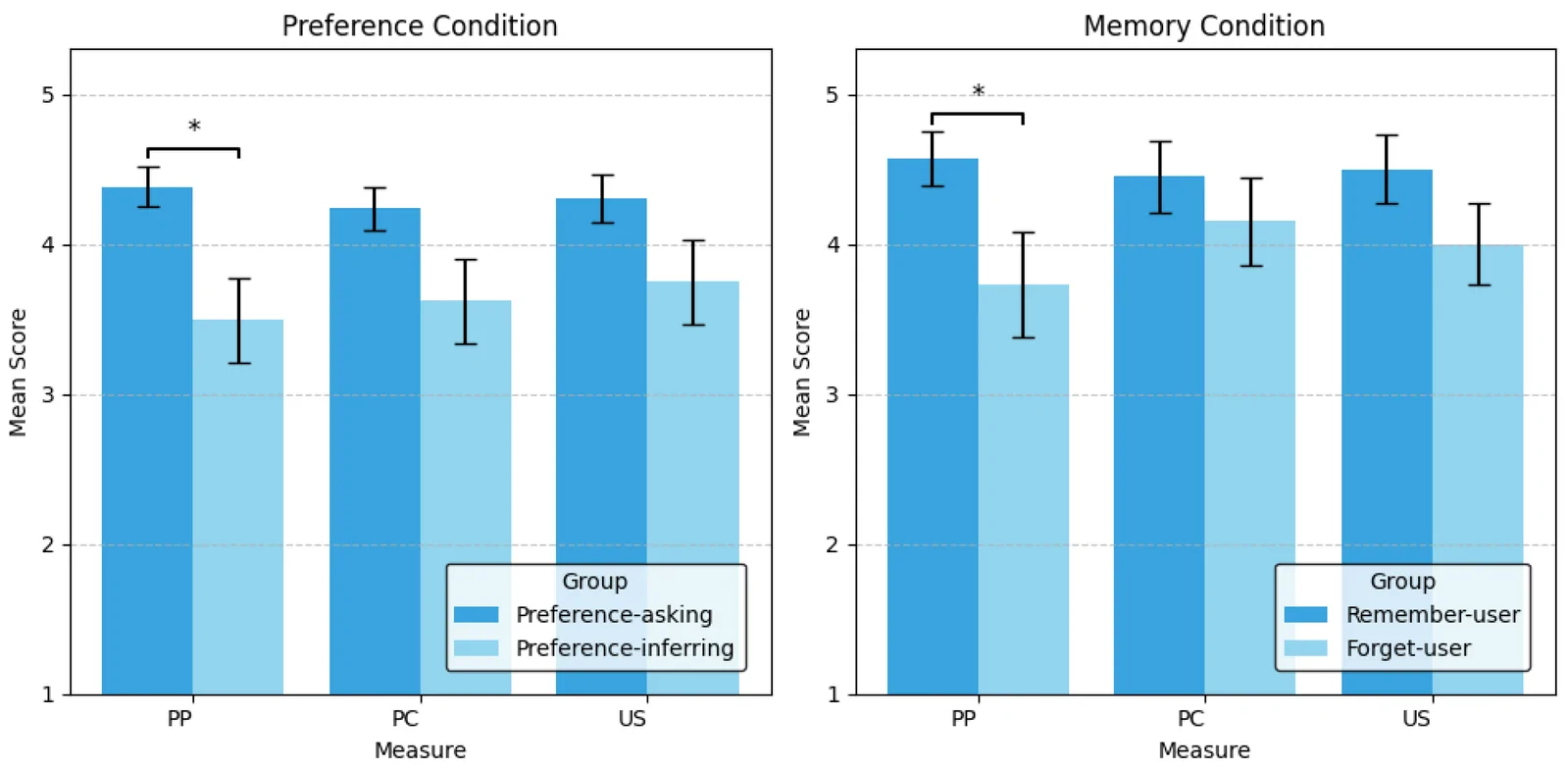
Mann-Whitney test results according to preference condition and memory condition (PP: Perceived Personalization, PC: Perceived Care, US: User Satisfaction; ANI: Animacy, ANT: Anthropomorphism, LIK: Likeability, INT: Perceived Intelligence, and SFT: Perceived Safety). * *p* < 0.05.

For perceived personalization, a statistically significant difference was found between Preference-asking group (*M* = 4.38, *SD* = 0.62) and Preference-inferring group (*M* = 3.49, *SD* = 1.30) (*U* = 128.500, *Z* = −2.349, *p* = 0.019), as depicted in Figure 6 (left). The rank-biserial correlation *R* was −0.363, indicating a medium effect size.

These results support Hypothesis 3, suggesting that when the robot delivered services without explicitly inquiring about user preferences (Preference-inferring group), the perceived level of personalization was significantly reduced compared to when preferences were solicited directly (Preference-asking group).

For perceived care, no statistically significant difference was observed between Preference-asking group (*M* = 4.24, *SD* = 0.66) and Preference-inferring group (*M* = 3.62, *SD* = 1.27) (*U* = 170.000, *Z* = −1.297, *p* = 0.195). The rank-biserial correlation *R* was −0.200, indicating a small effect. The manner in which the robot inquired about user preferences did not significantly influence participants’ perceptions of the robot’s attentiveness or emotional warmth.

Similarly, for user satisfaction, no significant difference was found between Preference-asking group (*M* = 4.30, *SD* = 0.73) and Preference-inferring group (*M* = 3.75, *SD* = 1.29), *U* = 173.500, *Z* = −1.206, *p* = 0.228. The rank-biserial correlation *R* was −0.186, reflecting a small effect size. The presence or absence of explicit preference inquiry had no statistically meaningful effect on participants’ overall satisfaction with the robot’s service.

#### 4.4.2. Personalization Through Memory of Past User Information

For perceived personalization, a statistically significant difference was observed between Remember-user group (*M* = 4.57, *SD* = 0.57) and Forget-user group (*M* = 3.73, *SD* = 1.11) (*U* = 22.000, *Z* = −2.159, *p* = 0.031), as illustrated in Figure 6 (right). The rank-biserial correlation *R* was −0.483, indicating a significant effect. These results support Hypothesis 4, demonstrating that perceived personalization was significantly lower when the robot failed to recall prior user information.

For perceived care, no statistically significant difference was found between Remember-user group (*M* = 4.45, *SD* = 0.76) and Forget-user group (*M* = 4.15, *SD* = 0.91) (*U* = 37.500, *Z* = −0.983, *p* = 0.326 (*R* = −0.220)). This suggests that recalling users’ prior information did not meaningfully affect how caring the robot was perceived to be. For user satisfaction, no statistically significant difference was observed between Remember-user group (*M* = 4.50, *SD* = 0.72) and Forget-user group (*M* = 4.00, *SD* = 0.85) (*U* = 31.500, *Z* = −1.455, *p* = 0.146 (*R* = −0.326)). This indicates that the robot’s memory of prior interactions did not significantly influence participants’ overall satisfaction with the service.

## 5. General Discussion

### 5.1. Integrated Interpretation of Personalization Cues

Across Studies 1–4, this research demonstrates that users nonetheless perceive personalization when a robot presents readily interpretable social cues. By examining docents’ adaptive service strategies (Study 1), robot performance competence (Study 2), knowledge alignment in explanation (Study 3), and explicit preference inquiry and memory-based continuity signals (Study 4), the findings collectively show that personalization arises not only from what the robot knows but from how it behaves within the interaction moment.

This study provides valuable implications for the design of human–robot interaction systems in public spaces by comparing the service behaviors of expert and novice science museum docents. The behaviors demonstrated by expert docents, such as sustaining eye contact to hold visitors’ attention using directive gestures to focus attention [37], and repositioning themselves to adapt to visitor groups [38], illustrate an understanding of social orchestration. These behaviors can be reframed as interaction strategies that consider the social context and can inform robot design elements such as gaze modeling, gesture timing, and spatial positioning. For instance, if a robot adjusts the duration of its eye contact according to the user’s attentional state, it may effectively reflect the engagement strategies of human docents [39].

Furthermore, service-level behaviors such as flexibility, proactivity, and interpersonal skills emerged as key features distinguishing the expert from the novice. The expert demonstrated flexibility by adjusting the complexity of explanations in real-time according to the visitor’s age and level of understanding, showed proactivity by anticipating and responding to visitors’ unspoken needs, and actively employed interpersonal skills to maintain conversational flow and interpret emotional cues. These behaviors highlight the need for robot design to move beyond repetitive, scripted responses toward interactions that are context-sensitive and responsive to user cues [40].

The interview analysis further clarified these differences. The docents who participated in the interview reported adjusting the difficulty and vocabulary of their explanations based on cues such as visitors’ ages, background knowledge, and verbal cues. For children, they avoided technical terms and incorporated more gestures to provide intuitive explanations, while for adults who asked technical questions, they shifted to more detailed explanations. In addition, they proactively responded to unspoken needs, such as guiding parents with infants to diaper-changing stations without being explicitly requested. These accounts show that expert service providers not only reacted to situations but also engaged in cognitive processes of reading the context and swiftly transforming their assessments into action.

However, how such behavioral differences are perceived within the actual user experience cannot be determined from the observation. Strategic docent behaviors may appear as contextual adjustments to an external observer. Still, to visitors, they may be interpreted as personalized experiences, such as “I was taken into consideration” or “the explanation was tailored to my level.” Thus, the more fundamental question can become: when do users perceive such adjustments not merely as service techniques but as personalization directed at them? The series of user studies, therefore, begun with this question, examining whether and how a robot’s behaviors can shape social impressions and foster users’ perceptions of personalized experiences.

Study 2 demonstrates that simply showing how well a robot performs a task can be sufficient for people to form social impressions of it. Users interpret a robot’s functional competence not merely as technical proficiency but as a basis for relational meaning [41]. In other words, perceptions of intelligence extend beyond evaluations of skill to broader impressions of the robot as a social entity, possessing agency and trustworthiness. Indeed, the participants evaluated the robot as more anthropomorphic, animated, and likable when it achieved higher recognition accuracy, despite the robot’s minimal use of behaviors such as gesture variability or prosodic modulation. This suggests that when people observe competent task performance, they may attribute intentionality and emotional qualities to the robot [42].

Interestingly, the recognition accuracy did not produce significant differences in the participants’ perceptions of safety. While a prior study [28] has suggested that higher performance can enhance psychological safety, the present findings imply that the recognition accuracy alone may be insufficient to shift safety perceptions in short, low-risk interactions. Instead, safety impressions appear to depend more heavily on embodied and contextual cues such as speed, physical appearance, and proximity.

Overall, the study suggests that basic functional reliability and the clear, straightforward presentation of tasks may constitute important principles in shaping user impressions in human–robot interaction. Even in the absence of complex gestures or expressive behaviors, a robot that reliably completes its tasks and communicates rules in an easily understandable manner can elicit perceptions of intelligence, likability, and human-likeness. This strategy may be particularly effective in public settings where interactions are brief and transient. Thus, leveraging functional performance itself as a social signal represents a practical design approach for human–robot interaction.

The other findings in Study 3 demonstrate that when a robot’s explanation aligns with the user’s prior knowledge, it can be perceived as a personalized experience. This is consistent with previous research showing that conceptual alignment in human–robot interaction can influence users’ linguistic behavior and induce cognitive-level changes [43]. In this regard, it has been argued that the central factor driving personalization is not merely algorithmic adaptation, but rather the extent to which the user perceives the interaction as personally relevant [44]. Our results similarly revealed that under identical explanatory conditions, the participants with an engineering background, whose knowledge aligned with the robot’s explanation, rated the interaction as significantly more personalized than those from non-engineering backgrounds. This suggests that epistemic resonance, defined as the cognitive match between explanatory content and prior knowledge, can create a sense of being personally addressed.

The participants’ descriptive responses further supported these quantitative differences. The engineering participants accurately referred to the robot’s internal mechanisms, demonstrating a higher level of conceptual integration. In contrast, the non-engineering participants relied more on metaphor and intuition, reflecting a more superficial understanding. The results of Study 3 reinforce this distinction, showing that personalization does not necessarily emerge from dynamic systems, but can also be sufficiently generated by static content that aligns with the user’s conceptual framework.

Study 4 examined two core mechanisms underlying perceived personalization in human–robot interaction: explicit preference inquiry and memory-based continuity. The findings demonstrate that the participants’ subjective perceptions of personalization are not determined solely by the robot’s technical capabilities but are instead shaped by how those capabilities are framed and enacted within the interaction [45].

The first significant finding is that directly asking users about their preferences significantly enhances perceived personalization. The participants who experienced explicit preference inquiry evaluated the robot’s service as more personally tailored. This result aligns with prior service research, which emphasizes that transparent interaction and user participation enhance the perceived value and satisfaction of service experiences [46]. Conversely, when the robot relied exclusively on internal inference without asking, perceived personalization was rated significantly lower. As shown in the results of Study 2, predictive accuracy is important in the social aspect, but personalization depends on communicative cues that acknowledge and relationally connect with the user. In other words, even systems with relatively simple architectures can elicit a strong sense of personalization when they are designed to actively incorporate and respect user participation [47].

A second key finding in Study 4 concerns memory. When the robot recalled and referenced prior interaction details, such as a user’s name or sticker preference, the participants rated personalization significantly higher than when such continuity was absent, minimal continuity signals, such as referencing past choices, enabled the participants to perceive the interaction as more personalized. The substantial effect size found in the memory condition (*R* = −0.483) underscores the psychological salience of continuity in interaction. Remembering and referencing even seemingly trivial details not only enhanced users’ perceptions of personalization but also suggested a sense of relational continuity, as users appeared to interpret such memory cues as signs of being personally recognized. Thus, appropriately surfacing minimal memory traces can significantly elevate users’ perceptions of a robot’s social competence and sophistication, even in short-term or one-off contexts such as museums [48].

It is noteworthy that the absence of preference inquiry or memory recall did not produce significant differences in participants’ perceptions of care or overall satisfaction. This suggests that perceived personalization operates as an independent dimension of the HRI experience. Users may feel cared for and satisfied with the interaction as long as the robot is polite and functionally competent. However, the sense of “being personally addressed” appears to arise only when the robot explicitly solicits preferences or recalls prior interactions. Finally, these findings are limited to the specific personalization strategies implemented in Study 4; different patterns may emerge in long-term interactions or with alternative forms of personalization.

Taken together, these results indicate that personalization impressions are shaped by micro-level social signals that require neither extensive user data nor complex adaptive algorithms. The following section discusses the psychological mechanisms grounded in CASA (Computers Are Social Actors) [49] and correspondence bias [50] that explain how such subtle cues produce rapid social attribution and the perception of personalization.

### 5.2. Social Attribution Mechanisms

The findings of this research demonstrate that the perception of personalization extends beyond the mere delivery of information and is mediated by micro-level social signals [51,52]. Small cues such as greeting the participants by name, adjusting the difficulty of explanations, recalling past choices, or explicitly asking about preferences created the impression of interacting with a considerate and responsive agent [53,54]. These effects were confirmed in Study 3 and Study 4, where experts perceived the use of technical terminology as a tailored explanation, and the simple act of referencing a prior sticker choice led to stronger perceptions of personalization.

Study 2, in contrast, offered a complementary but distinct insight. Here, the robot’s basic functional performance, specifically its visual recognition accuracy, was observed to translate into enhanced social impressions. When recognition accuracy was high, the participants rated the robot as more humanlike, likable, and intelligent. This illustrates a cognitive bias whereby users interpret technical success as an indicator of social intelligence. In other words, even functional performance may be construed as attentive behavior, thereby providing a psychological foundation for the perception of personalization. These results suggest that functional cues, such as recognition accuracy, and cognitive and relational cues, such as knowledge alignment and memory continuity, provide complementary perspectives on how users socially attribute personalization to robots.

Interestingly, the recognition accuracy did not produce significant differences in the participants’ perceptions of safety. While a prior study [55] has suggested that higher performance can enhance psychological safety, the present findings imply that the recognition accuracy alone may be insufficient to shift safety perceptions in short, low-risk interactions. Instead, safety impressions appear to depend more heavily on embodied and contextual cues such as speed, physical appearance, and proximity, suggesting that perceived safety may rely on a different class of cues than those that drive personalization. To further verify this effect, future work should systematically manipulate physical interaction parameters such as a robot’s speed, interpersonal distance, and body size to more precisely examine the interaction between functional performance cues and safety perceptions.

These phenomena can be explained through psychological theories. The CASA paradigm posits that humans automatically apply social scripts and interpersonal norms to nonhuman agents once minimal social cues are present [49]. In this view, cues such as accurate recognition, topic-appropriate wording, or memory references are interpreted not as pre-defined system operations, but as communicative behaviors directed at the user. These findings reflect CASA’s emphasis on cue combinations: task competence (Study 2), epistemic alignment (Study 3), and explicit preference inquiry and memory-based continuity (Study 4) represent complementary layers of cues that, from a theoretical perspective, can jointly enhance social attribution beyond what any single cue alone implies. This suggests that effective personalization in public service HRI benefits from bundling functional, cognitive, and relational cues within a single encounter, especially in brief and anonymous museum interactions. At the same time, correspondence bias explains why users infer stable internal dispositions and intentionality from a robot’s behavior, even when they are aware that such actions may be driven by simple rules or fixed scripts [50]. When a robot performs correctly, users attribute intelligence and attentiveness to the agent; conversely, errors may be interpreted as indicators of disinterest or incompetence. Thus, social attribution in HRI is not merely a reflection of technical output, but the result of human-centred inference about the robot’s intentions.

### 5.3. Design Implications

#### 5.3.1. Interaction Design for Social Adaptivity

To begin with, modeling the behaviors of human docents demonstrates the potential to systematically transfer their interaction strategies into robotic interactions. For instance, a docent’s gestures to direct attention toward a specific exhibit or the deliberate slowing of speech to gauge audience reactions represent intuitive strategies that can be incorporated into robot design. Empirical studies have already shown that techniques such as gestural anchoring, adaptive pacing, and audience-tailored speech enable museum docent robots to effectively capture visitor attention through simple head movements [56]. Such behaviors extend beyond functional task support; they serve as interpretive tools for managing audience attention, inferring levels of comprehension, and sustaining engagement. Translating these patterns into robotic actions makes it possible to simulate socially acceptable responsiveness even without genuine comprehension, thereby providing a foundation for a design framework of impression-driven adaptivity.

In addition, the finding that even minimal cognitive cues (e.g., visual recognition accuracy) trigger social attribution underscores the human tendency to interpret basic functionalities as evidence of social agency. Based on this result, one can envision a museum docent robot that, upon correctly recognizing an exhibit, might say, “This model was built in 1980 as an industrial robot for welding,” leading visitors to perceive it not only as functionally competent but also as attentive and intelligent. Likewise, if recognition errors occurred, a socially competent response such as, “I misrecognized that. Let me check again,” could transform the mistake into a signal of attentiveness. Such responses align with prior evidence showing that robot competence constitutes a core determinant of cognitive, affective, and behavioral trust in human–robot interaction [57]. Notably, these impressions emerged without enhancements to appearance, expressivity, or linguistic variation. Visitors might tend to interpret even simple performance signals as indicators of internal states, such as the robot paying attention. Consequently, competence and affective evaluation are inseparable in user interpretation, frequently blending. This effect suggests that, instead of relying solely on expressive augmentation, cognitive transparency offers an efficient pathway for designing socially competent robots.

Furthermore, among the mechanisms that support perceptions of personalization, knowledge alignment emerged as a representative method for embedding users’ cognitive frames into explanatory content [58]. This process involves tailoring explanations to the audience’s knowledge base or preferred communication style, thereby strengthening perceptions of personalization [59]. In contexts of one-off interactions, prior user profiling may not always be possible; instead, personalization can be achieved by allowing users to select the desired level of explanation themselves or by enabling the system to adjust content immediately based on situational cues. For instance, in a museum robot context, children might receive an intuitive and engaging explanation such as, “This robot can walk and dance like a person,” whereas domain experts might be provided with technical content such as, “This robot employs servo motors in its joints and uses a gyroscope for balance control.” Multilayered explanatory formats that allow the choice of both complexity and modality have been proposed, while proactive targeting strategies leveraging contextual cues such as typical visitor profiles at specific times of day to achieve epistemic resonance without individual identification have been emphasized. Integrating such approaches enables the delivery of content that feels personally tailored, even in brief, structured, and sometimes repetitive interactions.

Finally, another personalization manipulation examined in this research was asking for a choice and recalling prior selections. When the robot explicitly inquired about user preferences or recalled past encounters, the participants perceived the robot as recognizing them as unique individuals. These manipulations significantly increased personalization scores but did not automatically translate into higher social warmth or satisfaction. This finding highlights the conceptual distinction between personalization and social warmth [60]. The stronger impact of memory continuity compared to preference inquiry can be attributed to its relational implications. Although preference solicitation acknowledges users’ present choices, continuity cues indicate ongoing recognition of the same individual, which users interpret as relationship maintenance rather than a one-time adaptation. Thus, even small memory references (e.g., recalling a past sticker choice) elicit deeper personalization impressions. While both contribute to heightened engagement, they operate through different mechanisms [61]. In other words, even when a robot is judged as efficient and likable, it may still be perceived as impersonal unless it signals explicit adaptation to the individual user. From a design perspective, this highlights the importance of deliberately incorporating relational cues, such as referential memory and explicit preference confirmation, to foster the perception that this service is for me. In the context of a robot museum, these strategies may be implemented by enabling the docent robot to recall prior choices, for instance, reminding a child of the humanoid robot zone previously visited or adjusting recommendations after a family selects the hands-on program. Such mechanisms can be operationalized through simple prompts, such as “Would you like to hear an interesting story or a technical explanation?” or “Do you remember the humanoid robot you saw last time?” These cues explicitly signal recognition of individual interests, thereby enhancing perceived personalization and reinforcing the distinctiveness of the museum experience.

#### 5.3.2. Scalable Personalization Under Real-World Constraints

Building on these insights, the findings of this research offer concrete design implications for implementing scalable personalization in public service contexts. Beyond dynamic adaptation, designers may prepare pre-defined explanatory modes in advance, akin to scripted dialogues or behaviors proposed in prior HRI research, which can then be aligned with different cognitive frames [62]. Although real-time adaptation was not implemented in the present study, the results confirmed that the participants nonetheless perceived personalization when the robot communication and content were aligned with their cognitive frames and situational context [63,64].

In future applications, lightweight rule-based reasoning grounded in observable cues such as age or speech complexity may enable the provision of appropriately tailored explanations, even without full real-time adaptivity. Such an approach is particularly suited to high-turnover, time-constrained environments, offering a scalable and socially intelligent alternative to complete behavioral adaptation. However, real-world deployments of public service robots must contend with implementation barriers, including sensing reliability, network connectivity, crowding under variable visitor flows, maintenance constraints, and data governance restrictions on privacy and retention. To ensure operational feasibility under such conditions, personalization systems should be designed to degrade gracefully when sensing or recognition is limited and to prioritize lightweight, context-based reasoning over intrusive or computationally intensive long-term profiling.

While positive impressions of competence and attentiveness are desirable, users may over-generalize social intelligence from minimal cues. When a museum robot nods, maintains eye contact, or uses polite language, adults may assume it can monitor children’s safety or respond to emergencies, shifting responsibility away from themselves, even in urgent situations. Children, who readily anthropomorphize interactive agents, may especially believe the robot can protect or guide them if it uses phrases like “Don’t worry, I will keep you safe,” implying real hazard recognition. To prevent miscalibrated trust, museum service robots should explicitly signal their limitations (e.g., “I can only guide you to the exhibit”), employ clear safety warnings and staff alerts when risky behavior occurs, and use interaction scripts that reinforce human supervision (e.g., “Please make sure an adult checks for your safety”). These strategies help ensure that visitors, particularly children, do not misinterpret a friendly robot as a fully autonomous caretaker.

### 5.4. Limitations

Several avenues for future research remain. To begin with, while this research examined personalization, social warmth, and perceived intelligence as separate dimensions, a more comprehensive theoretical framework that integrates these constructs should be developed, offering an opportunity to systematically clarify conceptual overlaps. Furthermore, although the discussion highlighted the positive effects of surface-level social signals, such as memory cues or name usage, future research should carefully consider the ethical implications of potential over-interpretation or misplaced trust.

While this research emphasized the effects of personalization, it remains an important task to delineate its scope and boundary conditions in more complex task environments or across diverse user groups. Although the current sample size and analytic approach constrain broad generalizability, the internal validity of the design supports directional inference about how minimal task cues modulate social perceptions of robots. As Studies 2–4 were conducted in controlled laboratory environments with primarily young adult participants, their generalization to dynamic museum contexts may be limited. Nonetheless, the findings provide actionable insights for designing socially responsive behaviors in real-world public service robots and highlight the need to examine whether similar effects extend to children, families, and older adults.

## 6. Conclusions

This research examined how social robots can convey personalization in public cultural venues where long-term user data are rarely available. Across docent observation and controlled laboratory studies, the findings demonstrate that recognition accuracy enhanced perceptions of anthropomorphism, animacy, likability, and competence, while knowledge alignment, preference inquiry, and memory-based continuity selectively strengthened perceived personalization. These effects emerged from immediately interpretable social signals rather than resource-intensive personalization infrastructures. Theoretically, this study provides a unified empirical account of complementary personalization cues that shape social impressions in brief, anonymous interactions. Practically, it translates strategies observed in experienced docents into design principles for socially responsive and low-resource public service robots, capable of fostering engagement even within transient encounters.

Future research should refine the conceptual scope of personalization, examine variability across user groups, and ensure that personalization does not inadvertently foster overreliance or misplaced trust, particularly among vulnerable visitors.

## Figures and Tables

**Table 1:** Behavior coding scheme defining the service-level behaviors observed in docent interactions.

Category	Definition
Flexibility (F)	Behaviors that respond to changes in visitor demands or situational changes.
Proactivity (P)	Behaviors that attempt to identify or anticipate visitor needs or situational changes.
Interpersonal Skills (IS)	Behaviors that elicit responses from visitors through engaging actions (e.g., maintaining attention).

**Table 2:** Behavior coding scheme defining the basic actions used for interaction analysis.

Category	Subcategory	Definition
Gaze	Object (Gaze-O)	Looking at objects (e.g., exhibits, tools).
	Visitor (Gaze-V)	Looking around at the visitor(s), including any accompanying persons.
Point	Object (Point-O)	Pointing out the object.
	Visitor (Point-V)	Pointing out the visitor.
Move	Toward Object (Move-O)	Moving toward the object and stops.
	Toward Visitor (Move-V)	Moving toward the visitor and stops.
Talk	Object (Talk-O)	Talking about the object.
	Question (Talk-Q)	Asking a question to the customer.
	Answer (Talk-A)	Responding to the customer’s question.
	Unexpected situation (Talk-U)	Talking about special or emergent situations (e.g., emergencies, hazards).
	Backchannel (Talk-B)	Giving a brief verbal response to acknowledge the customer’s statement.

**Table 3:** A list of semi-structured interview questions for museum docents.

**Category**	**Interview Questions**
Background & Experience	Q1. How long have you been working as a museum docent? Q2. What do you consider most important when interacting with visitors?
Assessing Visitor Characteristics	Q3. How do you adjust explanation style based on age differences? Q4. How do you recognize visitors with prior technical knowledge? Q5. How does your explanation change for technically knowledgeable visitors?
Verbal & Non-verbal Adaptation	Q6. How do your gestures, gaze, and expressions change when interacting with children? Q7. What actions do you take when visitors lose attention? Q8. How do you decide when to point to exhibits or move during explanations?
Flexibility & Situational Responses	Q9. How do you maintain explanations when unexpected situations occur (e.g., robot malfunction)? Q10. Can you describe a case where you adjusted complexity spontaneously? Q11. How do you manage communication when multiple visitors are present?
Relational Personalization	Q12. What strategies do you use to personalize one-off interactions? Q13. Do you ever ask for visitors’ names or personal details? Under what circumstances?
Contextual & Spatial Adaptation	Q14. What types of personalization are possible in lobby or transition spaces? Q15. Does your approach change depending on the exhibition area or space?

## Data Availability

All data are available upon request.
